# Supplementary material for: Cancer Prevention: Knowledge, Attitudes and Lifestyle Cancer-Related Behaviors among Adolescents in Italy
Source: Int J Environ Res Public Health. 2020 Nov 10;17(22):8294. doi: 10.3390/ijerph17228294 (PMC7698075; doi:10.3390/ijerph17228294)
Supplement: Supplementary file 1 [file ijerph-17-08294-s001.pdf]

S1 SUPPLEMENTARY FILE

QUESTIONNAIRE

Section A. SOCIO-DEMOGRAPHICS

A1. Gender □ Male □ Female **A2.** Age ____ **A3.** Height______cm **A4.** Weight _____Kg

A5. What is the occupation of your father? ____________________

A6. What is the occupation of your mother?_____________________

A7. What is the highest education degree of your father? ________________________

A8. What is the highest education degree of your mother? _______________________

A9. Do you know anyone who has had cancer? □No □ Yes, who? (specify)_______________________

A10. Would you say your health is … □ Excellent □ Good □ Discrete □ Poor

Section B. KNOWLEDGE RELATED TO CANCER

B1. Cancer is a disease caused by uncontrollable cell growth and can spread to various parts of the body.

Have you ever heard of it? (more than one answer allowed) □No □Yes, by who? □Tv □ Internet

□ Physicians □ School/teacher □ Family □ Other (specify)___________________________

B2. On your opinion, which of the following factors can increase the chance of developing cancer?

	**Yes**	**No**	**I don't know**
Smoking	□	□	□
Passive smoking	□	□	□
Overweight/Obesity	□	□	□
Poor physical activity	□	□	□
Low fruit/vegetables intake	□	□	□
Daily intake of sweets and/or sugary drinks	□	□	□
High fat diets	□	□	□
Alcohol drinking	□	□	□
Sun exposure without sun protection	□	□	□
Use of tanning lamps	□	□	□

B3. On your opinion, at what age can cancers appear? (more than one answer allowed)

□ At any age □ After 18 years □ Between 18 and 65 years □ After 65 years

B4. On your opinion, which are the most frequent cancers in Italy? (more than one answer allowed)

□ Colorectal cancer □ Cancer of the uterus □ Prostate cancer

□ Liver cancer □ Breast cancer □ Stomach cancer □ Lung cancer

B5. On your opinion, which of the following cancers are associated with smoking? (more than one answer allowed) □ Lung cancer □ Oral cancer □ Esophageal tumor

□ Stomach cancer □ Colorectal cancer □ Bladder cancer

□ Ovarian cancer □ Leukemia

B6. On your opinion, which of the following cancers are associated with alcohol consumption? (more than one answer allowed)

□ Mouth and throat cancer □ Colorectal cancer □ Breast cancer □ Liver cancer

Section C. ATTITUDES RELATED TO CANCER

C1. How much worried are you about developing cancer? (1 not at all worried; 10 very worried)
1 2 3 4 5 6 7 8 9 10

C2. Cancers are serious diseases □ Agree □ Uncertain □ Disagree
C3. Cancers are preventable diseases □ Agree □ Uncertain □ Disagree

Section D. LIFESTYLE BEHAVIORS RELATED TO CANCER
D1. Do you currently smoke cigarettes? □ No (go to D3.) □ Yes
D2. In the previous month, how many days did you smoke cigarettes?
□ None □ 1-2 □ 3-5 □ 6-9 □ 10-19 □ 20-29 □ All 30 days
D3. In the last week, how many days did you do perform physical activity for a total of at least 60 minutes a day?
□ Never □ 1 day □ 2 days □ 3 days □ 4 days □ 5 days □ 6 days □ 7 days
D4. Generally, in your leisure time, how much time a day do you spend sitting, watching television, using a computer, playing videogames, or doing other activities while sitting?
□ Half an hour □ 1 to 2 hours □ 3 to 4 hours □ 5 to 6 hours □ 7 to 8 hours □ More than 8 hours
D5. How many times per day do you usually eat fruit?
□ Never □ 1 times per day □ 2 times per day □ 3 times per day □ 4 times per day □ 5 or more times per day
D6. How many times per day do you usually eat vegetables?
□ Never □ 1 times per day □ 2 times per day □ 3 times per day □ 4 times per day □ 5 or more times per day
D7. How many times per day do you consume sugary drinks?

□ Never □ 1 times per day □ 2 times per day □ 3 times per day □ 4 times per day □ 5 or more times per day

D8. How often do you consume alcoholic drinks?

□ Never □ Less than once a month □ Once a month □ 2-4 times a month □ 2-3 times a week

□ 4 or more times a week

D9. On the days you drink, how many alcoholic beverages do you consume on average?

□ 1-2 □ 3-4 □ 5-6 □ 7-9 □ 10 or more

D10. How often do you drink 6 or more drinks in a single occasion?

□ Never □ Less than once a month □ Once a month □ Once a week □ Every day or almost

D11. In the last six months have you had sun exposure without adequate protection (eg. sunscreen)? □ No □ Yes

D12. Have you used tanning lamps in the past six months? □ No □ Yes

D13. Have you had the Papillomavirus (HPV) vaccination? □ No □ Yes □ I don't know

Section E. INFORMATION

E1. Do you receive information on cancer prevention?

□ No □ Yes, from what sources? (more than one answer allowed) □ Family □ Physicians □ School/teacher □ Scientific journals □ Internet □ Other (specify) ____________________

E2. Would you like more to receive information on cancer prevention? □ No □ Yes

S2 SUPPLEMENTARY FILE

Variables included in the logistic regression models with related categories

MODEL 1: Good knowledge about risk factors for cancer

Independent variables	Code
Age, in years (ordinal)	13-15=1
	16-17=2
	18-20=3
Gender	male=0
	female=1
Both parents employed	no=0
	yes=1
Personal, familiar, or friend history of cancer	no=0
	yes=1
Physicians as sources of information about cancer prevention	no=0
	yes=1
Need of additional information about cancer prevention	no=0
	yes=1

MODEL 2: Being **c**urrent smokers

**Independent variables**	**Code**
Age, in years (ordinal)	13-15=1<br>16-17=2<br>18-20=3
Gender	male=0<br>female=1
Personal, familiar, or friend history of cancer	no=0<br>yes=1
Physicians as sources of information about cancer prevention	no=0<br>yes=1
Education level of at least one parent	lower than college degree=0<br>college degree or higher=1
At least one parent in the healthcare sector	no=0<br>yes=1
Having heard the definition of cancer from a physician	no=0<br>yes=1
Knowledge of the most frequent cancers in Italy and of those related to smoking and alcohol use	no=0<br>yes=1
Knowledge that insufficient intake of fruits/vegetables, daily intake of sweets and/or sugary drinks, and high fat diets are risk factor for cancer	no=0<br>yes=1
Knowledge that poor physical activity and overweight/obesity are risk factor for cancer	no=0<br>yes=1
Smoking is a risk factor for cancer	no=0<br>yes=1
Self-reported health status	unsatisfactory (<5)=0<br>satisfactory (5)=1
Perception of risk of developing cancer	low (1-7)=0<br>high (8-10)=1
Consuming alcohol	no=0<br>yes=1
Consuming at least 5 servings of fruits and vegetables per day	no=0<br>yes=1
Having a daily intake of sugary drinks	no=0<br>yes=1
Being physically active	no=0<br>yes=1

MODEL 3: Being physically active

Independent variables	Code
Age, in years (ordinal)	13-15=1<br>16-17=2<br>18-20=3
Gender	male=0<br>female=1
Personal, familiar, or friend history of cancer	no=0<br>yes=1
Physicians as sources of information about cancer prevention	no=0<br>yes=1
Education level of at least one parent	lower than college degree=0<br>college degree or higher=1
At least one parent in the healthcare sector	no=0<br>yes=1
Having heard the definition of cancer from a physician	no=0<br>yes=1
Knowledge of the most frequent cancers in Italy and of those related to smoking and alcohol use	no=0<br>yes=1
Knowledge that insufficient intake of fruits/vegetables, daily intake of sweets and/or sugary drinks, and high fat diets are risk factor for cancer	no=0<br>yes=1
Knowledge that physical activity and overweight/obesity are risk factor for cancer	no=0<br>yes=1
Smoking is a risk factor for cancer	no=0<br>yes=1
Self-reported health status	unsatisfactory (<5)=0<br>satisfactory (5)=1
Perception of risk of developing cancer	low (1-7)=0<br>high (8-10)=1
Consuming alcohol	no=0<br>yes=1
Consuming at least 5 servings of fruits and vegetables per day	no=0<br>yes=1
Having a daily intake of sugary drinks	no=0<br>yes=1
Being physically active	no=0<br>yes=1
Being current smokers	no=0<br>yes=1
